# Inflammasome sensor NLRP1 disease variant M1184V promotes autoproteolysis and DPP9 complex formation by stabilizing the FIIND domain

**DOI:** 10.1016/j.jbc.2022.102645

**Published:** 2022-10-26

**Authors:** Jonas Moecking, Pawat Laohamonthonkul, Kubilay Meşe, Gregor Hagelueken, Annemarie Steiner, Cassandra R. Harapas, Jarrod J. Sandow, Jonathan D. Graves, Seth L. Masters, Matthias Geyer

**Affiliations:** 1 Institute of Structural Biology, Medical Faculty, University of Bonn, Bonn, Germany; 2 Inflammation Division, The Walter and Eliza Hall Institute of Medical Research, Parkville, Australia; 3 Department of Medical Biology, University of Melbourne, Parkville, Victoria, Australia; 4 IFM Therapeutics, Boston, Massachusetts, USA

**Keywords:** NLRP1, inflammasome, DPP9, Talabostat, Val-boroPro, asthma, AF2, AlphaFold2, ASC, apoptosis-associated speck-like protein containing a CARD, CARD, caspase activation and recruitment domain, DPP9, dipeptidyl peptidase 9, FIIND, function-to-find domain, GST, glutathione-*S*-transferase, HEK293T, human embryonic kidney 293T cell line, IP, immunoprecipitation, MALS, multiangle light scattering, MBP, maltose-binding protein, MD, molecular dynamics, MW, molecular weight, NACHT, domain found in NAIP, CIITA, HET-E, and TEP1, nanoDSF, nano differential scanning fluorimetry, NLRP1, nucleotide-binding oligomerization domain–like receptor containing a pyrin domain 1, PYD, pyrin domain, SEC, size-exclusion chromatography, SPR, surface plasmon resonance, TCEP, Tris(2-carboxyethyl)phosphine, TEV, tobacco etch virus, Ub, ubiquitin, VbP, Val-boroPro

## Abstract

The inflammasome sensor NLRP1 (nucleotide-binding oligomerization domain–like receptor containing a pyrin domain 1) detects a variety of pathogen-derived molecular patterns to induce an inflammatory immune response by triggering pyroptosis and cytokine release. A number of mutations and polymorphisms of NLRP1 are known to cause autoinflammatory diseases, the functional characterization of which contributes to a better understanding of NLRP1 regulation. Here, we assessed the effect of the common NLRP1 variant M1184V, associated with asthma, inflammatory bowel disease, and diabetes, on the protein level. Our size-exclusion chromatography experiments show that M1184V stabilizes the “function-to-find” domain (FIIND) in a monomeric conformation. This effect is independent of autoproteolysis. In addition, molecular dynamics simulations reveal that the methionine residue increases flexibility within the ZU5 domain, whereas valine decreases flexibility, potentially indirectly stabilizing the catalytic triad responsible for autocleavage. By keeping the FIIND domain monomeric, formation of a multimer of full-length NLRP1 is promoted. We found that the stabilizing effect of the valine further leads to improved dipeptidyl peptidase 9 (DPP9)-binding capacities for the FIIND domain as well as the full-length protein as determined by surface plasmon resonance. Moreover, our immunoprecipitation experiments confirmed increased DPP9 binding for the M1184V protein in cells, consistent with improved formation of an autoinhibited complex with DPP9 in activity assays. Collectively, our study establishes a molecular rationale for the dichotomous involvement of the NLRP1 variant M1184V in autoimmune syndromes.

Inflammasome sensor proteins like the nucleotide oligomerization domain–like receptors NLRP1 (nucleotide-binding oligomerization domain–like receptor containing a pyrin domain 1), NLRP3, or NLRC4 are recognized as key components of the innate immune system (1, 2). The general concept of their mode of action is described as a sequence of events upon activation by specific triggers. These triggers include a range of molecular patterns and can be of endogenous (danger-associated) or exogenous (pathogen-associated) origin (3). Activation of the sensor protein induces conformational changes resulting in oligomerization and subsequent recruitment of the adaptor protein ASC (apoptosis-associated speck-like protein containing a caspase activation and recruitment domain [CARD]) and procaspase-1, forming an active inflammasome (4, 5). Within this protein complex, procaspase-1 is cleaved into its active form and can in turn cleave gasdermin D and proinflammatory cytokines interleukin-1β and interleukin-18 into their active forms, leading to pyroptosis and inflammation (3, 4, 6).

The inflammasome sensor proteins are typically comprised of an N-terminal effector domain, a central NACHT (domain found in NAIP, CIITA, HET-E, and TEP1) domain, and a number of C-terminal leucine-rich repeats. Recruitment of ASC is mediated *via* the N-terminal effector domain, which is a pyrin domain (PYD) for NLRP3 or a CARD for NLRC4 (3). Besides an N-terminal PYD, NLRP1 contains an additional “function-to-find” domain (FIIND) and CARD on its C terminus and is thus unique among NLRs (6, 7). The FIIND domain consists of two interwoven subdomains, called ZU5 and UPA, and undergoes autoproteolysis in between these two subdomains (8). The autolytic cleavage occurs on the peptide bond linking residues F1212 and S1213. To date, multiple studies found that for NLRP1, the C-terminal fragment (UPA–CARD) resulting from autoproteolysis is the inflammasome-forming part and thus responsible for ASC recruitment (9, 10, 11, 12). While autoproteolysis within the FIIND domain seems to be constitutive, the C-terminal cleavage fragment remains associated with the N-terminal domains through interactions between the ZU5 and UPA domains and is released only upon degradation of the N-terminal domains (13, 14, 15). Importantly, blocking autoproteolysis by mutating the cleavage site completely abrogates NLRP1 inflammasome formation (13). The current understanding is that degradation of the N-terminal fragment occurs at low rates during homeostasis but is drastically increased upon activation of NLRP1 (14, 15, 16).

A key component for keeping this system in check is dipeptidyl peptidase 9 (DPP9), which forms a complex with NLRP1 by binding one full NLRP1 molecule (N- and C-terminal fragments) and one free C-terminal cleavage fragment that inserts into the active site of DPP9 without being processed by the peptidase (16, 17, 18). By capturing free UPA–CARD fragments, it prevents oligomerization of these and therefore the formation of an active inflammasome. Multiple molecular triggers are described to activate NLRP1, like viral proteases, dsRNA, and ribotoxic stress (*e.g.*, induced by UVB irradiation) (19, 20, 21, 22, 23, 24, 25). All these triggers directly affect the N-terminal domains of the NLRP1 protein either by protein cleavage, direct binding, or induced phosphorylation, respectively. An exception to this is the DPP9 inhibitor Val-boroPro (VbP, also named Talabostat), which binds to the active site of the peptidase and consequently replaces the part of the UPA–CARD fragment that binds this site (16, 18). As a result, the C-terminal fragment of NLRP1 is released from the NLRP1–DPP9 complex and thus able to form an active inflammasome.

Among the known triggers leading to NLRP1 activation are also missense mutations found in patients presenting with symptoms of autoinflammation (9, 26, 27). On a molecular level, these mutations activate NLRP1 through different mechanisms. For instance, the A66V mutation in the N-terminal PYD leads to activation of NLRP1 by disrupting the domain fold (9). The P1214R mutation leads to activation by preventing the UPA domain from inserting into the active site of DPP9, prohibiting complex formation in a similar manner as VbP (16, 17, 26). The single-nucleotide polymorphism rs11651270 causes the missense mutation methionine 1184 to valine (M1184V) within the FIIND domain (13, 28). Functionally, this variant has been shown to have increased autoproteolysis activity in the FIIND domain and to affect the level by which NLRP1 can be activated depending on the stimulus (13, 28). Although this variant does not cause hyperactivation of NLRP1 *per se*, as autoproteolysis itself is required but not sufficient for NLRP1 inflammasome activation (28), it has been associated with an increased risk of developing several autoimmune syndromes such as asthma or Crohn's disease (28, 29, 30, 31).

Aiming to investigate the molecular basis for the observed effects of this variant, we sought to assess the impact of the M1184V mutation directly on the protein level. Interestingly, we found that this single amino acid substitution stabilizes a monomeric conformation of the FIIND domain and a multimeric conformation of full-length NLRP1. This effect translates to increased autoproteolysis and enhanced DPP9 binding. Functional analysis in cells revealed that this amino acid exchange increased the capacities of NLRP1 to form an autoinhibited complex with DPP9. Combining biochemical analyses, molecular modeling, and functional assays in cells, this work provides a molecular rationale for the functional consequences of the M1184V variant in NLRP1.

## Results

### NLRP1 variant M1184V prevents FIIND domain oligomerization

**Figure 1:**
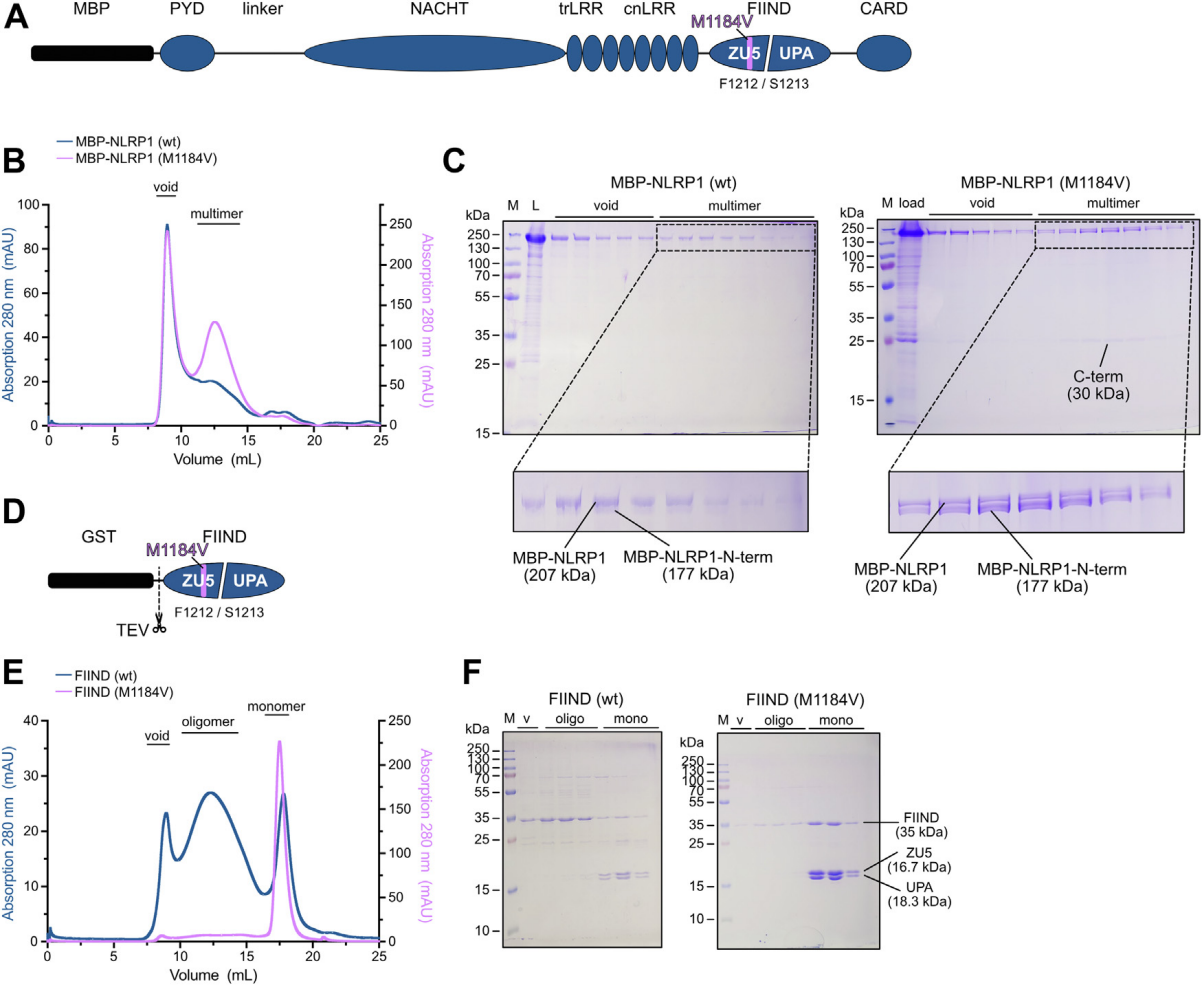
**NLRP1 variant M1184V prevents FIIND domain oligomerization.***A*, schematic of NLRP1 full-length expression construct with N-terminal MBP tag. Functional domains are depicted as PYD (pyrin domain), NACHT (domain found in NAIP, CIITA, HET-E, and TEP1), trLRR (transition leucine-rich repeats), cnLRR (canonical leucine-rich repeats), FIIND (domain with function to find), and CARD (caspase activation and recruitment domain). The two subdomains of the FIIND are labeled separately as ZU5 and UPA, and the location of the M1184V substitution is highlighted. *B*, elution profiles of MBP-NLRP1 wt and M1184V run on a Superose 6 size-exclusion column. *C*, SDS-PAGE of size-exclusion fractions shown in *B*. *D*, schematic of FIIND domain expression construct. The N-terminal GST tag is removed by TEV protease cleavage. *E*, elution profiles of NLRP1-FIIND wt and M1184V run on a Superose 6 size-exclusion column. *F*, SDS-PAGE of size-exclusion fractions shown in *D*. CT, C-terminal cleavage fragment; FIIND, function-to-find domain; GST, glutathione-*S*-transferase; L, load; M, molecular weight marker; MBP, maltose-binding protein; mono, monomer; NLRP1, nucleotide-binding oligomerization domain–like receptor containing a pyrin domain 1; NT, N-terminal cleavage fragment; oligo, oligomer; TEV, tobacco etch virus; v, void.

To date, the main observed difference between wt NLRP1 and the disease-associated M1184V mutant is the increased autoproteolysis within the FIIND domain (13). However, the molecular basis for this increase is still unknown. To gain a detailed understanding of the effects of this variant at the protein level, we designed expression constructs of full-length NLRP1 as N-terminal maltose-binding protein (MBP) fusion proteins (Fig. 1*A*). Both wt and mutant proteins were expressed in *Sf9* insect cells and purified *via* affinity pull down to homogeneity. Interestingly, in size-exclusion chromatography (SEC) experiments, the wt protein displayed a markedly dissimilar elution behavior compared with the M1184V variant protein. Full-length, wt MBP-NLRP1 eluted as a single peak in the void volume of the chromatography column, indicating a high molecular weight (MW) species (Fig. 1*B*). Only the right flank of this peak transitioned into a less pronounced second peak. All fractions spanning these two peaks contained the full-length NLRP1 protein as confirmed by SDS-PAGE analysis (Fig. 1*C*, *left panel*). Importantly, the protein appears almost completely as full-length variant and uncleaved from autoproteolysis. The second peak instead indicates the presence of the N-terminal cleavage fragment resulting from FIIND domain autocleavage. Based on the elution volume, the MW of the second peak suggests the existence of a defined multimer of NLRP1. For MBP-NLRP1 M1184V, this second peak was significantly more pronounced (Fig. 1*B*). Consistent with previous findings, the variant exhibited increased autoproteolysis activity (13). SDS-PAGE analysis of the corresponding elution fractions clearly showed that the second peak contained more of the cleavage fragments (Fig. 1*C*, *right panel*). The identity of all bands, the full-length, the N-terminal, and the C-terminal fragments, was confirmed by peptide mass fingerprint analysis (Fig. S1*A*).

To further corroborate that the observed effect of the M1184V variant on the oligomerization state of the full-length protein is a direct effect of this substitution, we focused on the FIIND domain itself. The FIIND was expressed in *Sf9* insect cells as N-terminal glutathione-*S*-transferase (GST) fusion protein, with the tag being removed by tobacco etch virus (TEV) protease cleavage before SEC analysis (Fig. 1*D*). Comparing SEC elution profiles of the wt and M1184V variant, a difference in the oligomerization state of the proteins was observed. While the wt protein eluted in three peaks (a void peak, an oligomer peak, and a monomer peak), the M1184V variant eluted almost exclusively as monomeric protein (Figs. 1*E*, S1, *B* and *C*). Analyzing the elution fractions revealed that the vast majority of the monomeric protein species of both the wt and variant FIIND protein appears as cleaved fragments (Fig. 1*F*). In contrast, the void and oligomeric species of the wt FIIND protein appear largely uncleaved. Off note, even the fully cleaved FIIND still runs as one protein in gel filtration experiments and not separated into its two entities. From these experiments, we conclude that the M1184V variant prevents oligomerization of the FIIND domain. For the full-length protein, this means that this variant is found less frequently in high MW fractions and is instead stabilized in a defined multimeric conformation. Interestingly, the multimeric species of the full-length NLRP1 protein and the monomeric species of the FIIND domain consist of mainly autocleaved protein. This prompted the question, whether autoproteolysis within the FIIND domain is the main driver of the observed differences in the multimeric assemblies between wt and M1184V variant protein.

### NLRP1 FIIND oligomerization prevents autoproteolysis

**Figure 2:**
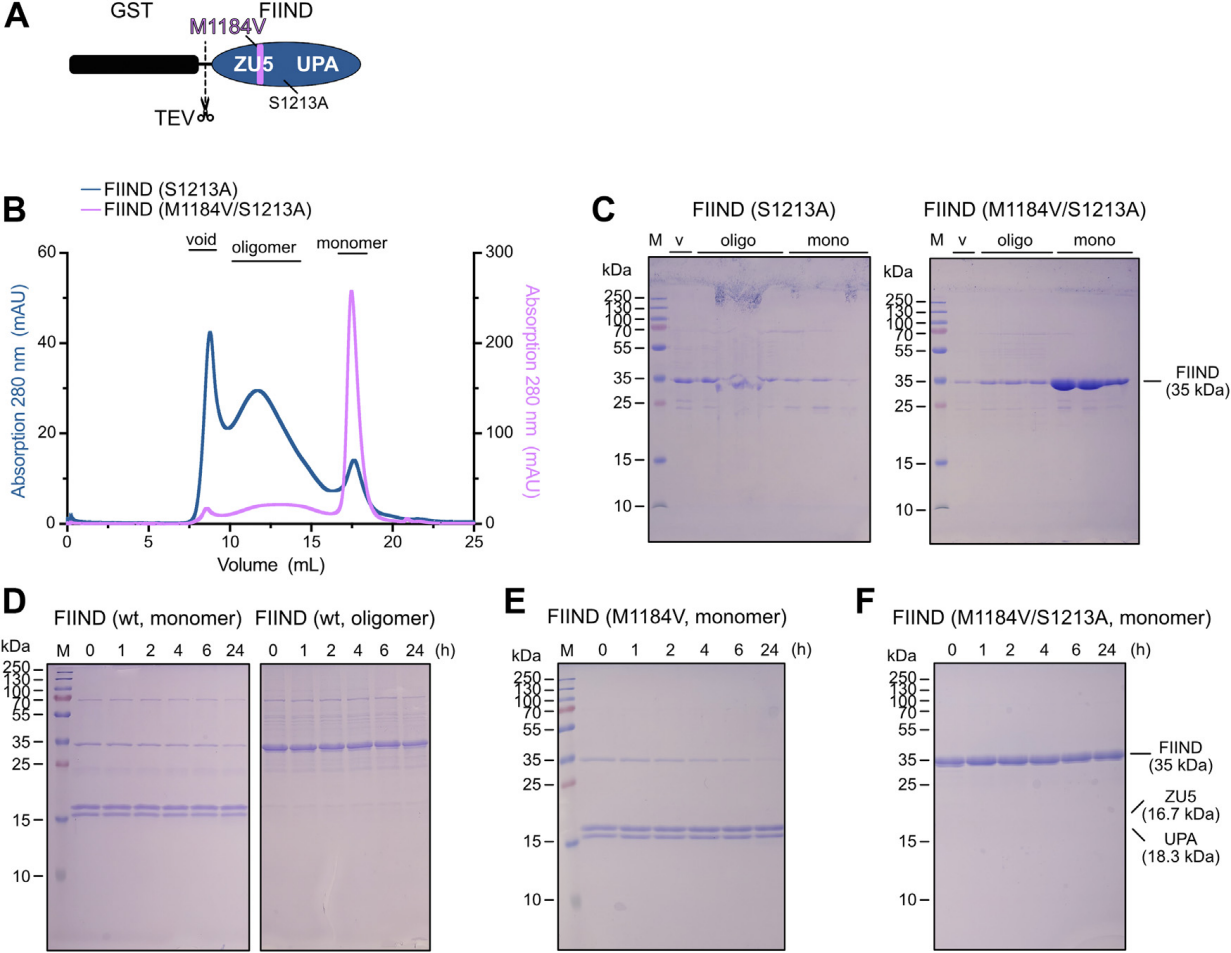
**NLRP1 FIIND oligomerization prevents autoproteolysis.***A*, schematic of the FIIND domain construct highlighting the S1213A mutation. *B*, elution profile of NLRP1 FIIND wt and M1184V combined with autoproteolysis inhibiting mutation S1213A run on a Superose 6 size-exclusion column. *C*, SDS-PAGE of size-exclusion fractions shown in *A*. *D*–*F*, incubation of different variants of purified FIIND domain protein (monomeric or oligomeric species) at 37 °C to assess autoproteolysis *in vitro*. About 2 μg of protein were sampled at indicated time points and analyzed by SDS-PAGE. Autoproteolysis is observed by a reduction in full-length FIIND (35 kDa band). FIIND, function-to-find domain; NLRP1, nucleotide-binding oligomerization domain–like receptor containing a pyrin domain 1.

To determine the effects of autoproteolysis on FIIND domain oligomerization, we introduced the S1213A mutation that completely abolishes proteolytic cleavage (Fig. 2*A*) (13). The elution behavior in SEC analysis of the S1213A mutant FIIND protein in context of the wt protein or the M1184V variant was directly compared (Fig. 2*B*). Overall, both elution profiles are similar to the cleavable version of the respective FIIND proteins (Figs. 1*E*, 2*B*, and S1*D*). Analysis of corresponding elution fractions by SDS-PAGE confirmed the presence of the FIIND domain in all observed peaks and the absence of any cleavage fragments (Fig. 2*C*). Thus, we conclude that the effect of M1184V to prevent oligomer formation of the FIIND domain is independent of autoproteolysis.

In SEC experiments with wt and M1184V FIIND protein, we noticed that only the monomeric but not the oligomeric FIIND protein peaks contained significant amounts of the cleavage fragments (Fig. 1, *E* and *F*). We therefore hypothesized that only FIIND monomers are capable of undergoing autoproteolysis. To test this hypothesis, different variants of purified FIIND protein were incubated at 37 °C for up to 24 h and subsequently analyzed by SDS-PAGE. For the monomeric species, a slight decrease of uncleaved FIIND for both wt and the mutant variant was observed over time, indicating that autoproteolysis is occurring under these conditions (Fig. 2, *D*, *left panel*, and *E*). In contrast, the oligomeric species of the wt FIIND protein did not show any decrease in uncleaved FIIND protein over time (Fig. 2*D*, *right panel*). The M1184V–S1213A autoproteolysis-deficient FIIND protein was used as a control and displayed no cleavage over time (Fig. 2*F*). This suggests that the increased autoproteolysis observed for the M1184V variant occurs because of its stabilizing effect on the monomeric conformation of the FIIND domain.

### NLRP1 M1184V reduces flexibility in the ZU5 domain

**Figure 3:**
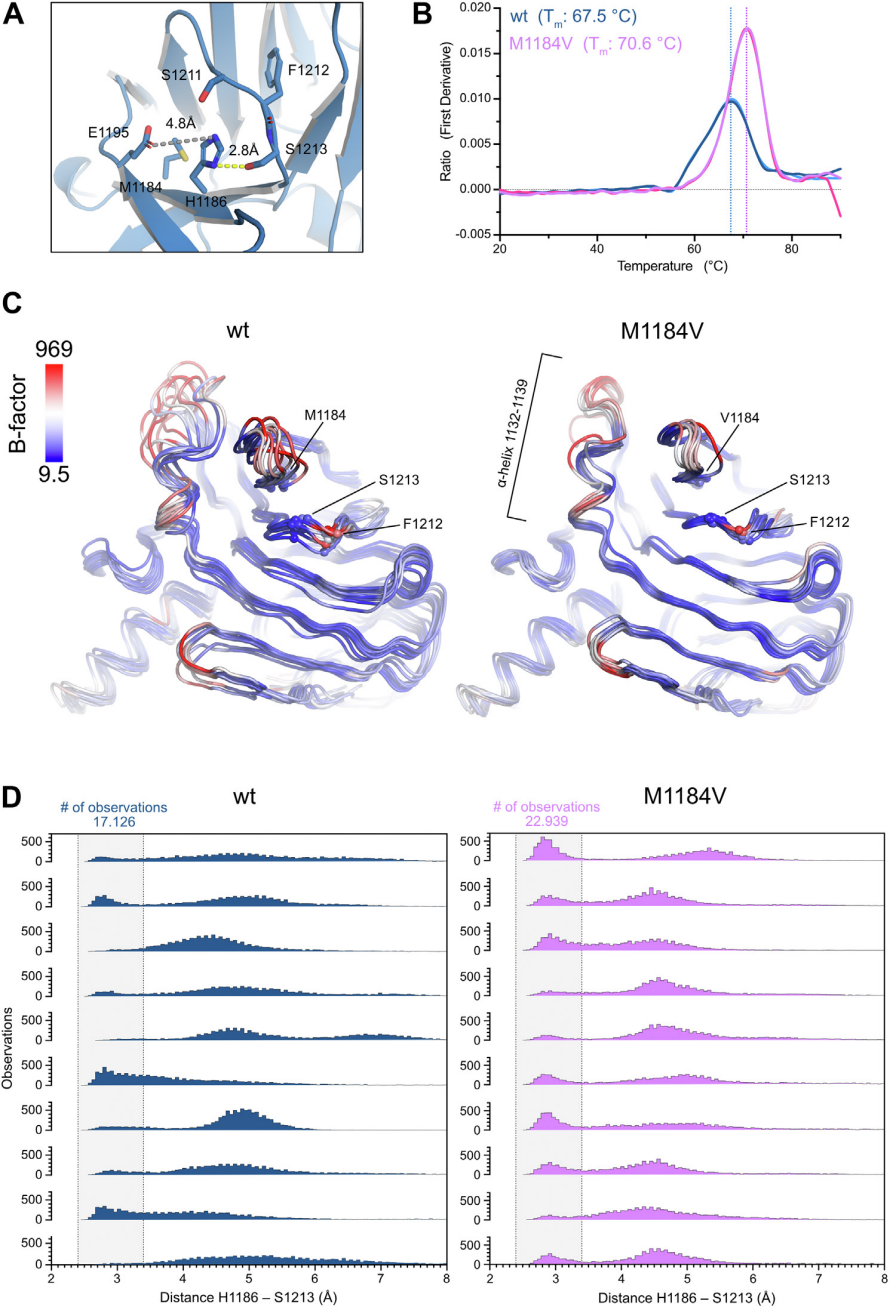
**M1184V variant reduces flexibility of the FIIND domain.***A*, depiction of the proposed catalytic triad required for autoproteolysis in the AF2 model of the wt FIIND domain. *B*, monomeric species of FIIND (wt or M1184V) were analyzed by nanoDSF. The temperature was increased from 20 to 90 °C at a rate of 1.5 °C/min. Melting temperatures were determined by calculating inflection points of the ratio of fluorescence signals recorded at 330 and 350 nm. The data are visualized as the first derivative of that ratio. Resulting *T*_m_s are indicated by *vertical dotted lines*. *C*, comparison of average *B*-factors over all molecular dynamics simulation. High *B*-factors are marked in *red*, and low *B*-factors are marked in blue. *D*, histograms of distances measured between the catalytic residues H1186 and S1213 based on 10 molecular dynamics simulation runs. *Gray bar* indicates range for potential hydrogen bond formation at a distance of 2.4 to 3.4 Å. AF2, AlphaFold2; FIIND, function-to-find domain; nanoDSF, nano differential scanning fluorimetry.

We next sought to assess the impact of the M1184V amino acid substitution on a structural level. As the data suggest that the described effect is largely independent of autoproteolysis, a model of the FIIND (amino acids 1064–1376) in its uncleaved state was generated using the AlphaFold2 (AF2) algorithm (Fig. S2*A*). Alignment to the existing structure of the rat NLRP1 FIIND (Protein Data Bank code: 7CRV) (18) resulted in an RMSD value of 0.958 Å for the wt FIIND model and 1.004 Å for the FIIND (M1184V) model indicating good conformity. As previously described, M1184 is located proximal to H1186, a critical residue of the catalytic triad consisting of E1195, H1186, and S1213 involved in autoproteolysis (Fig. 3*A*) (13, 28). As indicated by the RMSD values, using the same input sequence with M1184 replaced by valine had no significant effect on the overall conformation of the FIIND domain in the resulting AF2 prediction (Fig. S2*A*). Taking a closer look at the methionine residue, we hypothesized that it can potentially adapt different rotamer conformations and therefore increase flexibility within the autoproteolysis site. In line with that, the monomeric wt FIIND protein displayed a significantly lower *T*_m_ (67.5 °C) compared with the M1184V FIIND protein (*T*_m_: 70.6 °C) in thermal stability experiments using nano differential scanning fluorimetry (nanoDSF) (Fig. 3*B*). Off note, these *T*_m_s are unusually high for human proteins indicating the vast stability of the FIIND domain.

The protein stability was further analyzed by applying molecular dynamics (MD) simulations of the uncleaved FIIND. A total of 10 × 100 ns was simulated for the wt and M1184V variant FIIND domain. To get a quantitative estimate of differences between these two simulations, the resulting datasets were analyzed by comparing the average *B*-factor (average over 100 ns) and by comparing the measured distances between the δ1/ε2-nitrogen of H1186 and the γ-oxygen of S1213 for each simulated state (Figs. 3*A* and S2*B*). In support of the observations of the SEC analysis and nanoDSF results, simulation of the wt protein revealed an increased local flexibility in regions proximal to methionine 1184 (Fig. 3*C*). When comparing the average *B*-factor of all 10 MD simulations for each individual residue, similar differences are observed (Fig. S2*C*). Interestingly, the mean distance between the two catalytic residues was slightly larger for the wt protein (4.7 Å) compared with the mean distance measured for the M1184V FIIND domain (4.4 Å) (Fig. 3*D*). Consistently, the number of observations within a range suitable for potential hydrogen bond formation between H1186 and S1213 (2.4–3.4 Å) was higher for the M1184V variant (22,939 observations) than for the wt (17,126 observations). This analysis suggests that the observed differences between the wt and variant proteins are intrinsic to the FIIND domain. While not providing direct evidence, the reduced flexibility in the ZU5 domain and the resulting increase in autoproteolysis may hint toward a role for the ZU5 domain in keeping the UPA, and consequently the FIIND domain, in a monomeric conformation.

### NLRP1–FIIND oligomerization alters DPP9 binding kinetics

Multiple studies have recently demonstrated the importance of DPP9 in negatively regulating NLRP1 activity by directly binding to the FIIND domain (16, 17, 18, 32). Therefore, we compared the binding kinetics of DPP9 to monomeric and oligomeric FIIND proteins by surface plasmon resonance (SPR) spectroscopy. To this end, DPP9 was immobilized on a streptavidin sensor chip, and different variants of FIIND protein were injected to assess binding.

**Figure 4:**
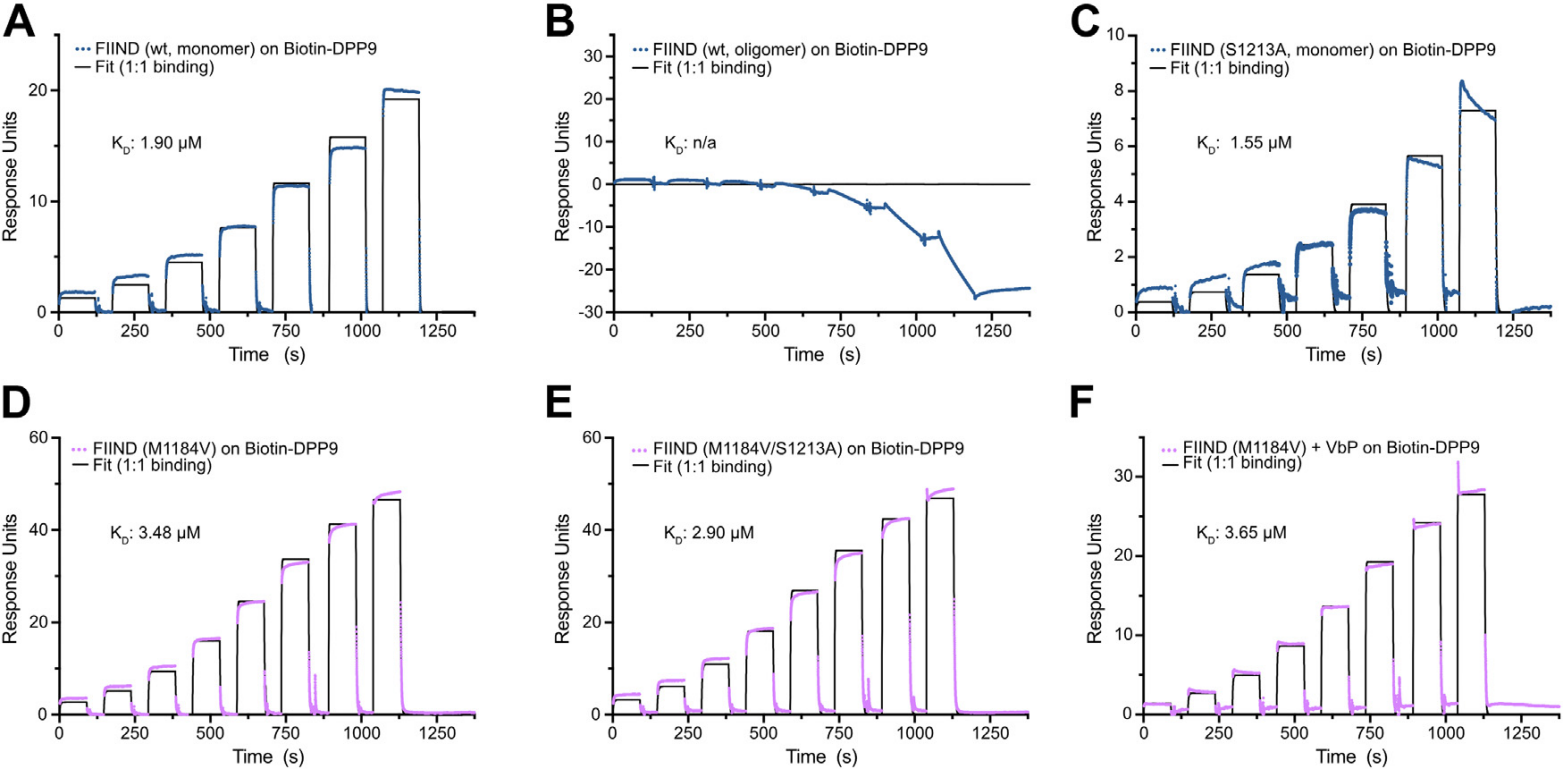
**Low-affinity binding to DPP9 is independent of autoproteolysis.***A* and *B*, single-cycle kinetic SPR measurements of binding of the monomeric and oligomeric forms of NLRP1-FIIND (wt) protein to immobilized biotin-DPP9. *C*, SPR analysis of autoproteolysis-deficient NLRP1-FIIND (S1213A) binding to DPP9. *D* and *E*, single-cycle kinetic SPR measurement of binding of NLRP1-FIIND (M1184V or M1184V S1213A) to immobilized biotin-DPP9. *F*, the measurement in *D* was repeated with 100 μM Val-boroPro (VbP) present in all dilutions of the FIIND domain. DPP9, dipeptidyl peptidase 9; FIIND, function-to-find domain; N/A, not applicable; NLRP1, nucleotide-binding oligomerization domain–like receptor containing a pyrin domain 1; SPR, surface plasmon resonance.

These SPR experiments showed that the monomeric fraction of the wt FIIND protein was able to bind DPP9, albeit with rapid association and dissociation kinetics. Together with the dissociation constant (*K*_*D*_) of 1.9 μM, this indicates a moderate affinity interaction with a strong electrostatic component (Fig. 4*A*). In contrast, the oligomeric fraction of the wt FIIND protein displayed unspecific binding to the reference, leading to a negative response in single-cycle kinetic measurements (Fig. 4*B*). Although a quantitative assessment of binding of the oligomeric FIIND protein to DPP9 was proven difficult in this SPR setting, the observed unspecific binding indicates an altered behavior of this protein species. As a control, GST showed no binding to DPP9 (Fig. S3*A*). Introducing the S1213A mutation to prevent autoproteolysis did not significantly alter binding for the monomeric species of the FIIND domain with a *K*_*D*_(S1213A) of 1.55 μM (Fig. 4*C*). Interestingly, even the P1214R mutation, reported to reduce DPP9 binding, still showed similar binding kinetics and affinity for the monomeric species with a *K*_*D*_(P1214R) of 3.43 μM (Fig. S3, *B*–*E*) (16, 28). This establishes that only the monomeric FIIND is capable of efficiently binding DPP9 and that this binding is independent of autoproteolysis and the presence of any free C-terminal UPA domain.

Next, we investigated whether the variant M1184V has a direct effect on the affinity of FIIND monomers for DPP9. The FIIND M1184V variant showed similar affinities for DPP9 with *K*_*D*_s of 3.5 μM for M1184V and 2.9 μM for M1184V–S1213A (Fig. 4, *D* and *E*). As for the wt protein, the interaction kinetics showed fast association and dissociation rates, indicative of a short-lived binding event between the FIIND domain and DPP9. The presence of the DPP9 inhibitor VbP or the P1214R mutation did not significantly affect the interaction to DPP9. Here, the *K*_*D*_ for the M1184V variant in the presence of 100 μM VbP is 3.65 μM, whereas the *K*_*D*_ of the M1184V–P1214R double mutation is 4.5 μM (Figs. 4*F* and S3*F*). Based on these results, we concluded that DPP9 binding is not directly affected by the M1184V amino acid exchange. Instead, our data suggest that increased DPP9 binding by this variant described in previous reports is due to the indirect effect of stabilizing a monomeric fold of the FIIND domain.

### Oligomerization of FIIND wt persists in the presence of DPP9

**Figure 5:**
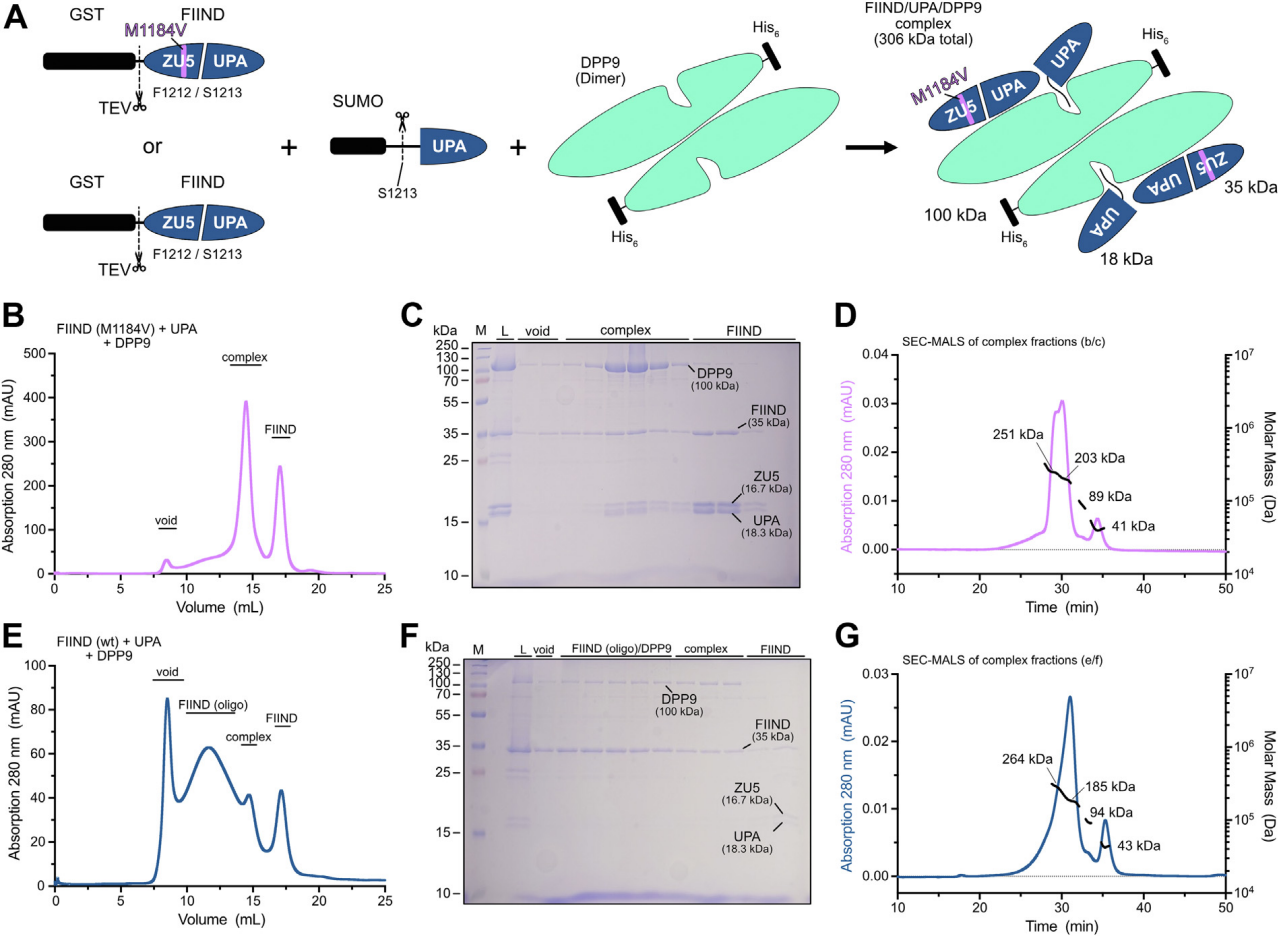
**NLRP1–DPP9 complex formation requires monomeric FIIND and free C-term fragment.***A*, schematic of NLRP1-FIIND and DPP9 constructs used in coexpression experiments including a SUMO-UPA construct for generation of a free UPA. The SUMO tag of the UPA construct is removed by endogenous SUMO proteases, and the GST tag of the FIIND is removed by TEV protease cleavage. Coexpression of the FIIND domain, DPP9, and UPA provides all required components for formation of the FIIND–UPA–DPP9 complex. *B*, elution profile of NLRP1-FIIND (M1184V) coexpressed DPP9 and a SUMO-UPA construct run on a Superose 6 size-exclusion column. *C*, SDS-PAGE of the elution fractions from *B*. *D*, SEC–MALS analysis of copurified NLRP1-FIIND (M1184V), DPP9, and UPA. *E*, elution profile of NLRP1-FIIND (wt) coexpressed with DPP9 and SUMO-UPA run on a Superose 6 size-exclusion column. *F*, SDS-PAGE of the elution fractions from *E*. *G*, SEC–MALS analysis of copurified complex of NLRP1-FIIND (wt), DPP9, and UPA. DPP9, dipeptidyl peptidase 9; FIIND, function-to-find domain; GST, glutathione-S-transferase; L, load; M, molecular weight marker; NLRP1, nucleotide-binding oligomerization domain–like receptor containing a pyrin domain 1; SEC–MALS, size-exclusion chromatography coupled with multiangle light scattering; TEV, tobacco etch virus.

We next sought to investigate whether the presence of DPP9 during the expression of the FIIND protein can prevent its oligomerization. In the *Sf9* cell expression system, no endogenous DPP9 or a homolog thereof is present. Therefore, the expression system was adapted to include a DPP9 construct being coexpressed in the same culture (Figs. 5*A* and S4*A*). Optionally, a construct producing a free UPA domain was included in the expression system, as previous structural studies showed that NLRP1–DPP9 complex formation requires full-length NLRP1 and a free C-terminal cleavage fragment (16, 18). First, we assessed if these coexpression systems allow complex formation between NLRP1 FIIND and DPP9. Therefore, NLRP1 FIIND M1184V was pulled down *via* its N-terminal GST tag and subsequently cleaved off from the beads by TEV protease. DPP9 binding was analyzed by gel filtration and SDS-PAGE. Independent of coexpression of free UPA, significant amounts of DPP9 were present after GST pulldown, and coelution of the FIIND and DPP9 proteins was observed in SEC analysis (Figs. 5, *B*, *C*, S4, *B*, and *C*). To further assess the stoichiometry of the coeluted FIIND–DPP9 protein complex, the according fractions from gel filtration were analyzed by SEC–multiangle light scattering (MALS). Here, only in the presence of additional free UPA, a tripartite complex of FIIND–UPA–DPP9 was observed, represented in a double peak of the SEC–MALS elution profile. The MW of 251 kDa calculated from the light scattering data from this peak is consistent with a complex consisting of a DPP9 dimer (200 kDa) with only one DPP9 molecule bound to a FIIND (35 kDa) domain and UPA (18 kDa) domain (Fig. 5*D*). Similar complexes were described in a previous structural study (18). Without the additional construct producing the free UPA, SEC–MALS analysis revealed a single peak with a MW of 192 kDa, indicative of a DPP9 dimer (Fig. S4, *D* and *H*). This would suggest that the ZU5 and UPA domains remain tightly bound, so that no significant amount of free UPA is available for complex formation. Consistent with that, coexpression of a cleavage-deficient FIIND mutant (M1184V–S1213A) with DPP9 gave a similar result (Fig. S4, *E*–*G*). Furthermore, the absence of a peak for a FIIND–UPA–DPP9 complex in the experiment without free UPA suggests that the FIIND–DPP9 complex slowly dissociates over the course of the SEC experiment. In support of that, a small additional peak with a calculated MW corresponding to monomeric FIIND is observed in each of the SEC–MALS runs (Figs. 5, *D*, *G*, S4, *D*, and *G*).

Based on the aforementioned results, we investigated the behavior of the wt FIIND protein in the presence of DPP9 and free UPA. As for the M1184V variant protein, significant amounts of DPP9 were pulled down (Fig. 5, *E* and *F*). However, the SEC elution profile still resembles the profile observed for the wt FIIND protein, with the additional oligomeric species. To our surprise, DPP9 coeluted with the oligomeric species as well as the monomeric species, demonstrating that the oligomeric FIIND protein still seems to be capable of DPP9 binding. Again, the SEC fractions corresponding to the FIIND–UPA–DPP9 complex were further analyzed by SEC–MALS (Fig. 5*G*). Similar as for the M1184V variant, this protein eluted mainly as a double peak, although the peak separation is significantly less pronounced. The MW for the left part of this peak was determined to 264 kDa, indicating a DPP9–FIIND–UPA complex with a similar stoichiometry as described previously. This demonstrates that both the FIIND wt and M1184V variant protein can form a complex with DPP9 and UPA. However, despite availability of DPP9 and free UPA during expression, oligomerization of the wt FIIND protein persisted. Ultimately, this led us to conclude that the observed oligomerization of the FIIND domain is independent of the presence of DPP9, strongly suggesting that the effect of the M1184V variant is entirely intrinsic for the FIIND domain.

### High MW NLRP1 species do not bind DPP9 efficiently

**Figure 6:**
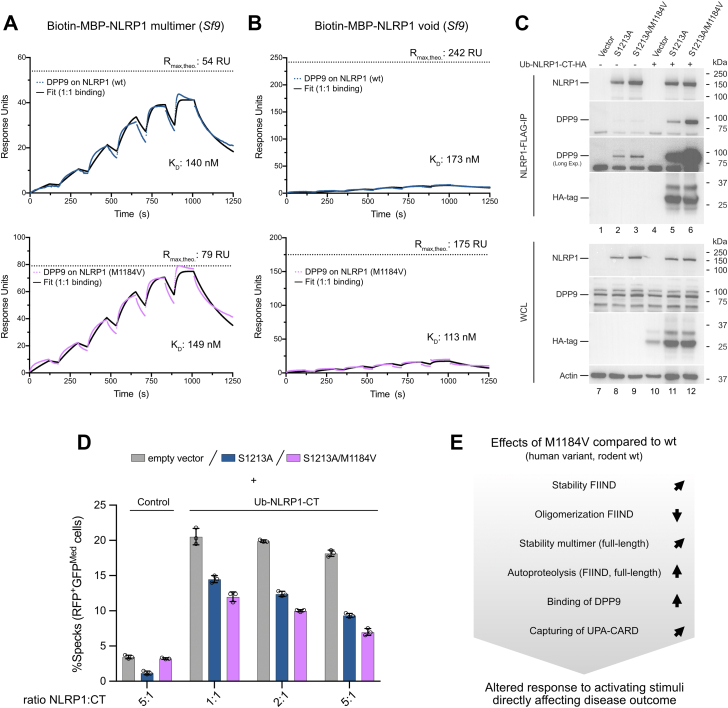
**NLRP1 M1184V favors DPP9 complex formation *in vitro* and in cells.***A* and *B*, SPR analysis of DPP9 binding to immobilized full-length biotin-MBP-NLRP1 (wt or M1184V) purified from *Sf9* cells and isolated from either the (*A*) NLRP1 multimer fraction or the (*B*) high MW (void) fraction. *C*, coimmunoprecipitation of endogenous DPP9 with NLRP1-FLAG (S1213A or M1184V/S1213A) in the absence or the presence of free NLRP1 C-term. The free C-term is generated by expression of a ubiquitin-NLRP1 (1213–1473, Ub-NLRP1-CT-HA) construct with a C-terminal HA tag. The ubiquitin is removed by endogenous proteases. *D*, ASC speck assay with NLRP1 C-term cotransfected with full-length NLRP1 (S1213A or M1184V/S1213A). Shown are data points from n = 3 independent experiments reported as mean ± SD values. *E*, schematic summary of effects of the M1184V variant on the protein level. ASC, apoptosis-associated speck-like protein containing a caspase activation and recruitment domain; DPP9, dipeptidyl peptidase 9; HA, hemagglutinin; MBP, maltose-binding protein; MW, molecular weight; NLRP1, nucleotide-binding oligomerization domain–like receptor containing a pyrin domain 1; *R*_max,theo_, theoretical maximum response at given immobilization level; SPR, surface plasmon resonance; WCL, whole-cell lysate.

As for the FIIND constructs, the M1184V variant showed an effect on the elution behavior of the full-length protein, preventing the formation of higher MW species (Fig. 1*B*). For the FIIND domain, differences between the monomeric and oligomeric species were observed with regard to their DPP9 binding (Figs. 4 and 5). Therefore, we asked if a similar effect can be observed for full-length NLRP1. First, we assessed DPP9 binding by SPR analysis. Biotin-FLAG-NLRP1 purified from human embryonic kidney 293T (HEK293T) cells or biotin-MBP-NLRP1 purified from *Sf9* cells was immobilized on a streptavidin-functionalized sensor chip, and DPP9 was injected at increasing concentrations (78–2500 nM). DPP9 showed binding to wt and M1184V protein from both expression systems with *K*_*D*_s of wt_HEK_ 155 nM, wt_*Sf9*_ 140 nM, M1184V_HEK_ 144 nM, and M1184V_*Sf9*_ 149 nM, revealing similar binding affinities throughout (Figs. 6*A* and S5*A*). While high MW MBP-NLRP1 (void) protein still showed DPP9 binding, the proportion of active protein (protein available for binding) on the SPR chip was significantly reduced to about 5 to 10% compared with the NLRP1 multimer (80–100% active). This effect was similar for both the wt and the M1184V protein (Fig. 6*B*). In contrast, the addition of VbP in the experiments did not significantly affect the binding, and DPP9 showed no binding to a biotin-MBP control protein (Fig. S5, *B* and *C*). These observations suggest that for full-length NLRP1, similar as for the FIIND domain, M1184V does not directly impact DPP9 binding. Instead, it indirectly enhances DPP9 binding by favoring the formation of a defined multimer of NLRP1 that binds DPP9 more efficiently than the high MW species.

### NLRP1 M1184V favors DPP9 complex formation in cells

DPP9 binding was further compared by coimmunoprecipitation (co-IP) of NLRP1-FLAG (S1213A or S1213A/M1184V) and endogenous DPP9 from HEK293T cells. Consistent with the biochemical data, DPP9 binding is enhanced for the NLRP1 S1213A/M1184V variant compared with NLRP1 S1213A (Fig. 6*C*, lanes 1–3). Coexpression of free C-terminal cleavage fragment with a ubiquitin (Ub) tag (Ub-NLRP1-CT-HA) generally increased the amount of DPP9 in the IP fraction. Still, NLRP1 S1213A/M1184V pulled down significantly more DPP9 compared with NLRP1 S1213A (Fig. 6*C*, lanes 4–6). In combination with the SPR data on the full-length NLRP1 protein, this suggests that NLRP1 wt produces increased amounts of high MW species incapable of DPP9 binding. In contrast, NLRP1 with the M1184V substitution is stabilized in the multimer conformation capable of DPP9 binding.

Finally, we aimed to assess consequences of the observed increase in NLRP1–DPP9 complex formation of the M1184V variant on NLRP1 activation in HEK cells. A construct encoding only the C-terminal UPA–CARD fragment with an N-terminal Ub tag (Ub-NLRP1-CT) was transfected into a HEK293T ASC-red fluorescent protein reporter cell line. The Ub is cleaved by intracellular proteases to release the native N terminus of the C-terminal fragment. In addition, an empty control vector or a construct encoding full-length NLRP1 containing the S1213A or S1213A/M1184V mutations was cotransfected at different ratios. As expected, expression of just the C-terminal fragment of NLRP1 induced robust ASC speck formation (Fig. 6*D*). Coexpression of the full-length NLRP1-FLAG mutants reduced ASC speck formation for both variants by capturing the C-terminal fragment in a complex with DPP9 and thereby preventing UPA–CARD filament formation (Fig. 6*D*). In agreement with increased complex formation with the C-terminal fragment and DPP9, the M1184V variant had a stronger inhibitory effect for all ratios examined. These functional experiments confirm that the stabilization of an NLRP1 multimer mediated by the M1184V variant is beneficial for the formation of the autoinhibited NLRP1–UPA–CARD–DPP9 complex.

## Discussion

Many studies with a focus on genetic risk factors in human NLRP1 describe SNP rs11651270, causing the missense mutation M1184V, to have dichotomous effects on the development of a multitude of autoimmune syndromes like asthma, inflammatory bowel disease, malignant melanoma, or diabetes (28, 31, 33, 34). This mutation has been shown to increase autoproteolysis in the FIIND domain of NLRP1, which is required but not sufficient for NLRP1-mediated inflammasome activation (13). On a functional level, NLRP1 M1184V displays diverse effects on inflammasome activation compared with the wt protein (28). However, the underlying molecular mechanisms are not well understood.

In this study, we demonstrate that the M1184V mutation directly affects NLRP1 protein assembly, leading to the stabilization of a multimeric NLRP1 complex. This is mediated directly through the FIIND domain, as it is retained in a monomeric state with a valine in position 1184. These effects are independent of autoproteolysis as demonstrated by introducing the S1213A mutation. Compared with the valine mutant, MD simulations indicate higher conformational flexibility for the region around the wt methionine at position 1184, which also spreads to a proximal helix (amino acids 1132–1139) in the ZU5 domain. At the same time, in accordance with previous experiments and our studies presented here, the NLRP1 M1184V variant displays increased autoproteolysis compared with wt NLRP1 (12, 27). These contrasting observations may be part of the puzzle surrounding the significance of this SNP, as the wt protein displays decreased autoproteolysis but may fall apart more easily, whereas the M1184V mutant is cleaved more efficiently but may hold together more tightly. A schematic summarizing the effects of the M1184V disease variant on the protein level is shown in Figure 6*E*.

We were intrigued by the initial observation that the MBP-NLRP1 (M1184V) mutant is able to form a large oligomer that is already seen in rudimentary form in the wt protein (Fig. 1*B*). This observation seems reminiscent to the recent description of a large oligomeric assembly of autoinhibited NLRP3, which forms decameric to hexadecameric assemblies larger than 1 MDa in size (35, 36). Stabilization of an autoinhibited state by the M1184V variant in the full-length NLRP1 multimer would be consistent with the preferential DPP9-binding capabilities this variant exhibits in SPR and co-IP experiments. Interestingly, a recent study also showed formation of DPP9-independent autoinhibited conformations of NLRP1 (37). Binding of the UPA domain to the linker region between the PYD and NACHT domain mediates this autoinhibition. Considering that the M1184V variant stabilizes the FIIND domain itself, the observed multimer of the full-length protein could be a direct result of this as it might be favorable for UPA binding to the linker region. Consequently, the NLRP1 multimer could also represent a partially DPP9-independent autoinhibitory conformation, which would, however, require further investigation.

The FIIND domain alone retains the ability to oligomerize but only in the uncleaved form (Figs. 1*E* and 2). While oligomerization is usually attributed to the NACHT domain in NLRs, or was recently shown to be mediated by the leucine-rich repeats (35, 36), the FIIND domain was shown to dimerize by the UPA domain in the 2:1 NLRP1 to DPP9 complex assembly (16, 18). In its activated state, the C-terminal UPA–CARD fragment forms helical filaments in which dimeric UPA domains spirally wrap as a ring-like oligomer around the inner CARDs (10, 11). In the FIIND domain assembly, the ZU5 domain is thought to block such interaction through steric hindrance, thus negatively regulating NLRP1 activation by inhibiting the formation of UPA–CARD filaments (18). Our data complement this functional description in that FIIND must be autolytically cleaved for the ZU5 domain to prevent oligomerization.

Multiple studies have pointed out differences in terms of DPP9-binding capabilities and inflammasome activation by different triggers between human and rodent NLRP1 variants (20, 24, 38, 39). The absence of a PYD in mouse Nlrp1 and major amino acid changes in the linker region preceding the NACHT domain are usually cited as explanations for these differences. Of note, rat and mouse NLRP1, but not human NLRP1, were previously described to bind DPP9 even in the absence of any cleavage fragments, also shown by mutation of the catalytic residues of the autoproteolysis site (17). For human NLRP1, only very weak binding to DPP9 was described for cleavage-deficient mutants, as assessed by IP (38). Sequence alignments show that rat and mouse Nlrp1 contain a valine in the position corresponding to human M1184 (13, 28). Thus, one possible explanation for the different DPP9-binding capacities of uncleaved NLRP1 and differences observed in the response to activating triggers in different species might be this single amino acid exchange.

Taken together, this study provides a mechanistic basis of how the M1184V variant stabilizes the FIIND domain, resulting in formation of an NLRP1 multimer and an increase in autoproteolysis (Fig. 6*E*). We further demonstrate how this effect translates into enhanced DPP9 binding, ultimately resulting in an increased capacity to retain free C-terminal cleavage fragments in an autoinhibited complex and reduce inflammasome activation in ASC speck assays. These mechanistic insights provide a molecular basis for the diverse effects this common NLRP1 variant has in the development of autoinflammatory diseases and advance our understanding of the mechanisms governing NLRP1 autoinhibition and activation.

## Experimental procedures

### Plasmids and constructs

Codon-optimized full-length human NLRP1 (UniProt accession number: Q9C000, isoform 1, residues 1–1473) was cloned into a modified pACEBac1 vector to be expressed as N-terminal MBP-tev fusion protein. The FIIND domain constructs (residues 1064–1376) were cloned into a pACEBac1 vector with an N-terminal GST-tev affinity tag. For NLRP1 biotinylation, full-length NLRP1 and BirA sequences were cloned into a modified pFastBac-Dual vector in which NLRP1 was fused to an Avi-MBP-tag encoding sequence in multiple cloning site 1. The BirA sequence was cloned into multiple cloning site 2 without any tag for coexpression. Point mutations were introduced by QuikChange mutagenesis. The sequence of all constructs and respective mutations was confirmed by sequencing prior to transfection.

### Protein expression and purification

All constructs were expressed in *Sf9* insect cells by infecting 500 ml of cells (2 × 10^6^ cells/ml) with 5% V2 baculo virus. The expression culture was harvested 48 to 72 h postinfection. The cells were collected by centrifugation (400*g*, 20 min), and the pellet was washed once in cold PBS and directly used for purification or frozen in liquid nitrogen and stored at −80 °C. For purification of recombinant full-length MBP-tev-NLRP1, the cell pellet was resuspended in buffer A (25 mM Hepes [pH 7.5], 150 mM NaCl, 0.5 mM Tris(2-carboxyethyl)phosphine [TCEP], and 10% glycerol) freshly supplemented with 10 μg/ml DNase I and 100 μM PMSF. Cells were lysed by sonication (30% amplitude, 5 min, pulse: 5 s ON/5 s OFF) on ice. The cell lysate was clarified by centrifugation at 75,000*g* for 45 min (10 °C). Afterward, the supernatant was filtered through an 0.45 μm syringe filter and applied to a 5 ml MBPTrap column at a flow rate of 2 ml/min using an ÄKTA prime chromatography system. After washing with at least 100 ml of buffer A, the bound protein was eluted in buffer A supplemented with 10 mM maltose. SEC of the eluted protein was run on a Superose 6 increase (10/300) GL column at a flow rate of 0.5 ml/min. Peak fractions were analyzed by SDS-PAGE, and fractions containing pure protein were collected, concentrated in an Amicon ultracentrifugation tube, snap frozen in liquid nitrogen, and stored at −80 °C until further use.

Purification of the GST-tev-FIIND and His_6_-tev-DPP9 constructs was carried out as described previously (18, 40). SEC was performed using a Superose 6 increase (10/300) GL column at a flow rate of 0.5 ml/min. Coexpression with DPP9 was achieved by coinfection with equal amounts of virus of each construct (GST-tev-FIIND constructs and His_6_-DPP9). Proteins from coexpressions were purified by pulling down the respective NLRP1 construct as described above.

### SPR spectroscopy

SPR spectroscopy measurements were carried out using a Biacore 8K system (Cytiva) as recently published (41). Before any measurement, the system was cleaned using the Desorb function. Data collection rate was 10 Hz for all immobilization and measurement steps. The measurement buffer consisted of 10 mM Hepes (pH 7.5), 150 mM NaCl, 0.5 mM TCEP, and 0.05% Tween-20. Prior to ligand immobilization, the chip was flushed three times with 1 M NaCl in 50 mM NaOH for 60 s at a flow rate of 10 μl/min. Intracellularly biotinylated Avi-His_6_-tev-DPP9 (25 nm) or Avi-MBP-tev-NLRP1 (5 nm) was immobilized on a Series S SA chip (Cytiva) at a flow rate of 2 μl/min for 600 s or 1200 s, respectively. Afterward, the system was washed with 50% 2-propanol in 1 M NaCl and 50 mM NaOH 50%. The wash step was followed by a stabilization time of 1200 s.

All measurements were run as single cycle kinetics with at least six different concentrations of analyte at a flow rate of 30 μl/min. The contact time was set to 120 s and the dissociation time to at least 240 s. In the case of DPP9 as the analyte, the concentrations applied in the kinetic measurement were 78.125, 156.25, 312.5, 625, 1250, and 2500 nM. For NLRP1-FIIND binding kinetics to immobilized DPP9, the same concentrations were used, but one additional concentration (5000 nM) was included. Only for the S1213A variant of the FIIND domain, a concentration of 39.06 nM was used as the lowest concentration, because of low protein yield.

### SEC coupled with MALS

Protein samples to be measured in SEC coupled with MALS were diluted to 0.5 to 1.0 mg/ml in a volume of 70 μl of buffer A without glycerol. About 50 μl were injected using an autosampler onto a Superose 6 increase (10/300) GL column connected to a 1260 Infinity HPLC system (Agilent), running with a flow rate of 0.5 ml/min. The outlet of the column was directly connected to an Optilab T-rEX refractive index detector following a miniDAWN MALS system (Wyatt). Setting baselines and defining peak areas were carried out using the ASTRA software (Wyatt). MW was determined based on the previous assignment in the ASTRA software.

### Mass spectrometry

Protein that was intended to be analyzed by mass spectrometry was separated by SDS-PAGE, and the gel was stained with SimplyBlue SafeStain (Thermo Fisher Scientific). Bands of interest were excised from the gel and transferred to a clean 1.5 ml polypropylene tube. Gel pieces were processed following a standard in-gel digest procedure. Prior to analysis, samples were lyophilized to dryness and stored at −80 °C. Samples were reconstituted in 20 μl of 0.1% formic acid and 2% acetonitrile before measurements. Samples were analyzed on an M-Class UHPLC (Waters) coupled to a timsTOF Pro (Bruker) mass spectrometer equipped with a CaptiveSpray source. Peptides were separated on a 25 cm × 75 μm Aurora analytical column, 1.6 μm C18 beads with a packed emitter tip (IonOpticks). The column temperature was maintained at 40 °C using an integrated column oven (Sonation GmbH). Samples were separated at 400 nl/min using a gradient from 2% to 17% buffer B (99.9% acetonitrile and 0.1% formic acid; 55 min), 17% to 25% buffer B (21 min) before ramping to 35% buffer B (13 min), then to 85% buffer B (3 min), and sustained for 10 min. The timsTOF Pro was operated in parallel accumulation–serial fragmentation mode using Compass Hystar 5.0.36.0 (Bruker). All raw files were analyzed by MaxQuant software using the integrated Andromeda search engine. Experiment type was set as trapped ion-mobility spectrometry–data-dependent acquisition with no modification to the default settings. Data were searched against the human UniProt Reference Proteome, which included the protein sequences expressed from the constructs, and a separate reverse decoy database using a strict trypsin specificity allowing up to two missed cleavages. The minimum required peptide length was set to seven amino acids. Modifications—carbamidomethylation of Cys was set as a fixed modification; N-terminal acetylation of proteins and oxidation of M were set as variable modifications. First search peptide tolerance was set at 20 ppm, and main search was set at 6 ppm (other settings left as default). Matching-between-runs and relative label-free quantitation was turned on. Maximum peptide mass was set at 8000 Da. All other settings in group or global parameters were left as default.

### Structural modeling and MD simulations

A structural model of the wt and M1184V variant FIIND domain in its uncleaved state was generated using the AF2 algorithm on an in-house server (42). Distance measurements and images were created using PyMOL (www.pymol.org). Explicit solvent MD simulations were performed with GROMACS 2021 using the OPLS-AA/L force field and the SPC/E water model (43, 44). The AF2 models were immersed in a dodecahedral box filled with water and ∼125 mM NaCl, such that the net charge of the system was neutralized. The system was equilibrated at 310 K for 100 ps with v-rescale temperature coupling and the pressure equilibrated to an atmospheric level for another 100 ps using the Parinello–Rahman barostat. For both the wt and M1184V variant, 10 independent 100 ns MD simulations were carried out with 2 fs time steps. The simulation results were analyzed with GROMACS and PyMOL.

### Cell culture

HEK293T cells were cultured in a humidified incubator at 37 °C and 5% CO_2_ in Dulbecco’s modified Eagle’s medium (Thermo Fisher Scientific) supplemented with 10% fetal bovine serum, 0.1% (w/v) streptomycin, and 100 U/ml penicillin. Fetal bovine serum was heat inactivated for 30 min at 55 °C before supplementation. Continuous cultures were monitored for the absence of mycoplasma by PCR.

### IP

About 2.5 × 10^5^ HEK293T cells were plated overnight before cotransfection with 500 ng of wt or mutant pCIG2-hNLRP1-3xFLAG-IRES-eGFP (S1213A or M1184V/S1213A) and 500 ng of empty vector (pcDNA3.1) or Ub-NLRP1-CT-HA. About 18 h post-transfection, cells were washed once with 1× Dulbecco’s PBS and harvested in NP-40 lysis buffer (1% NP-40 [v/v], 10% glycerol [v/v], 20 nM Tris–HCl, 150 mM NaCl, 1 mM EGTA, 10 mM NaPP_i_, 5 mM NaF, 1 mM Na_3_VO_4_, and 1 mM PMSF) freshly supplemented with 1× cOmplete protease inhibitor cocktail (Roche). After lysing cells for 20 min on ice, cell debris was removed by centrifugation, and the supernatant was collected. IP was performed using anti-FLAG-M2-agarose resin (Sigma) for 4 h or overnight at 4 °C. Beads were washed three times with lysis buffer before elution by boiling in SDS sample buffer for 10 min. Immunoblots were prepared using 4 to 12% gradient gels (Novex, Invitrogen) and subsequently transferred to a polyvinylidene difluoride membrane. Membranes were blocked in PBS/Tween-20 with 5% skim milk for 60 min at room temperature and probed overnight at 4 °C. Antibodies: α-NLRP1: AL176 (AdipoGen), α-DPP9: ab42080 (Abcam), α-FLAG: 9H1 (in house), and α-actin: sc47778 (SCBT).

### ASC speck formation assay

About 5 × 10^4^ HEK293T cells stably expressing human ASC-red fluorescent protein were plated in a 24-well plate overnight before cotransfecting with increasing concentrations of autoproteolysis-deficient pCIG-hNLRP1-3xFLAG-IRES-eGFP S1213A or M1184V/S1213A and an empty vector (pcDNA3.1) or Ub-NLRP1-CT-HA. Cells were harvested and analyzed for speck formation 12 to 16 h post-transfection by flow cytometry.

### NanoDSF

*T*_m_ determination by nanoDSF was carried out using a Prometheus NT.48 (NanoTemper) device. FIIND protein samples were set up in duplicates with a concentration of 10 μM in buffer containing 10 mM Tris–HCl (pH 8.0), 100 mM NaCl, and 0.5 mM TCEP. Fluorescence at 330 and 350 nm was recorded over a temperature range from 20 to 90 °C with a temperature increase of 1.5 °C/min. For analysis, the first derivative of the ratio of the two fluorescence signals (330 nm/350 nm) was calculated. The resulting peak indicates the apparent *T*_m_.

## Data availability

All data in this study are available within the article, supporting information, and/or from the corresponding author on reasonable request.

## Supporting information

This article contains supporting information. Mass spectrometry analyses of NLRP1 and cleavage fragments are shown in Fig. S1. AF2 modeling and the strategy of MD simulations are presented in Fig. S2. SPR data of the FIIND P1214R mutation on immobilized DPP9 are provided in Fig. S3. Fig. S4 shows the SEC analysis of NLRP1 FIIND coexpressed with DPP9, and SPR control measurements of DPP9 binding to MBP-NLRP1 and MBP are displayed in Fig. S5.

## Conflict of interest

M. G. received funding from IFM Therapeutics. S. L. M. receives funding from NRG Therapeutics and Odyssey Therapeutics. All other authors declare that they have no conflicts of interest with the contents of this article.
