# Supplementary material for: Inflammasome sensor NLRP1 disease variant M1184V promotes autoproteolysis and DPP9 complex formation by stabilizing the FIIND domain
Source: J Biol Chem. 2022 Oct 26;298(12):102645. doi: 10.1016/j.jbc.2022.102645 (PMC9700037; doi:10.1016/j.jbc.2022.102645)
Supplement: Supporting Information [file mmc1.pdf]

Supporting Information

Inflammasome sensor NLRP1 disease variant M1184V promotes autoproteolysis and DPP9 complex formation by stabilizing the FIIND domain

Jonas Moecking, Pawat Laohamonthonkul, Kubilay Meşe, Gregor Hagelueken, Annemarie Steiner, Cassandra R. Harapas, Jarrod J. Sandow, Jonathan D. Graves, Seth L. Masters, and Matthias Geyer

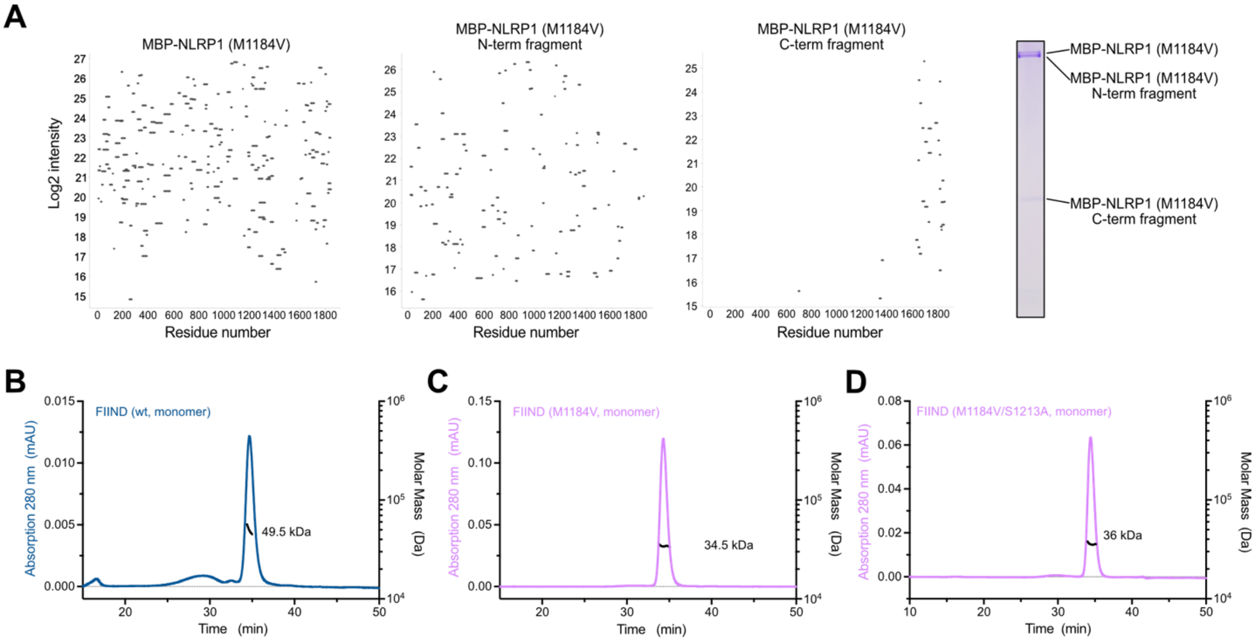

Figure S1. Biophysical analysis of recombinant full-length NLRP1 and recombinant NLRP1-FIIND. *A*, SDS-PAGE of MBP-NLRP1 M1184V multimer protein highlighting the different fragments resulting from autoproteolysis. All three bands were subjected to LC-MS/MS analysis. Visualized is the peptide coverage based on the MBP-NLRP1 fusion construct (residues 1-1853 including MBP). *B–D*, SEC-MALS analysis of monomeric FIIND protein species. The FIIND (wt or M1184V) protein sample was run on a Superose 6 column and analyzed by MALS directly afterwards. The molecular weight of 34.5 kDa and 36 kDa determined by light scattering confirms the monomeric state of the FIIND (M1184V) and FIIND (M1184V/S1213A) protein, respectively. The slightly increased molecular weight of 49.5 kDa of the FIIND (wt) protein might be due to slight oligomerization resulting from concentrating the sample.

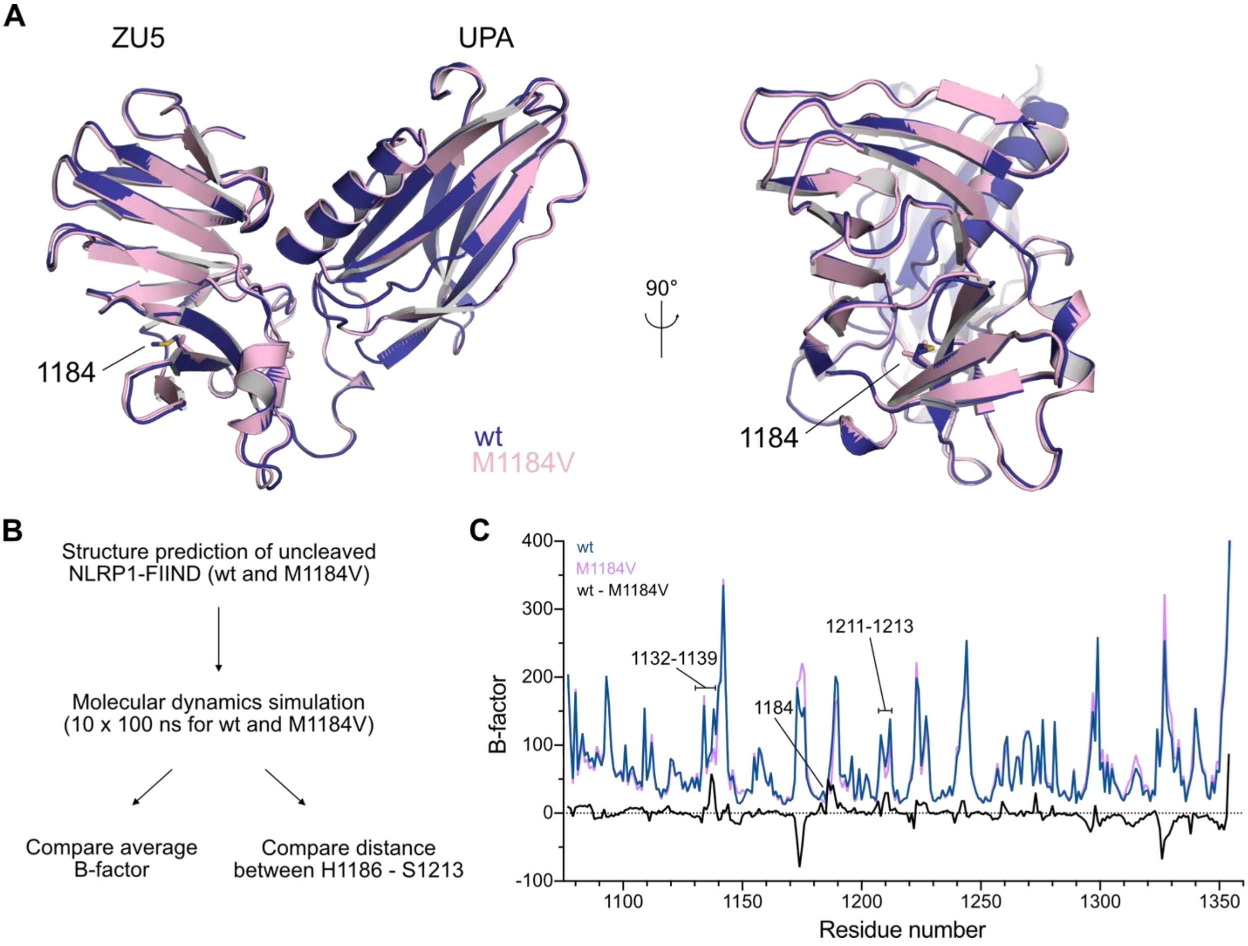

Figure S2. Modelling and in silico analysis of uncleaved NLRP1 FIIND.
A, AlphaFold2 prediction of the NLRP1-FIIND domain in its uncleaved form. The sequence of human NLRP1 (wt or M1184V) covering residues 1026-1376 were used for the prediction (sequence based on Uniprot ID: Q9C000-1). *B*, The generated models were applied to molecular dynamics simulations as described in the flow chart. Simulations were run for 100 ns with 2 fs steps. *C*, Average B-factor for each individual residue (average over 10 individual molecular dynamics simulations). To highlight differences between the resulting B-factors, the subtracted values (average B-factor (wt) – average B-factor (M1184V)) are plotted (black line).

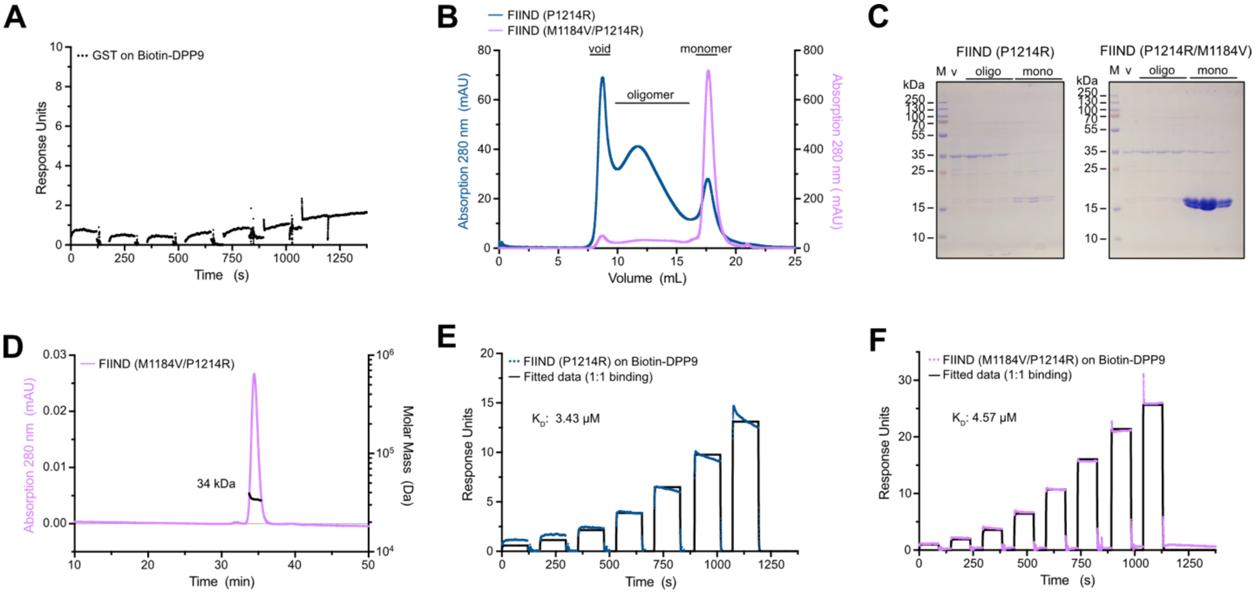

Figure S3. Biochemical characterization of the FIIND P1214R mutant.
A, Single cycle kinetic measurement of GST on immobilized Biotin-DPP9. GST was injected at the same concentrations as the FIIND protein. *B*, SEC elution profile of the P1214R and M1184V/P1214R mutant FIIND domains. *C*, SDS-PAGE of the elution fractions of the mutant FIIND domains. *D*, SEC-MALS analysis of the M1184V/P1214R mutant FIIND domain. The molecular weight of 34 kDa determined by light scattering confirms the monomeric state of the protein. *E*-*F*, Single cycle kinetic SPR measurement of the mutant FIIND domains on immobilized Biotin-DPP9.

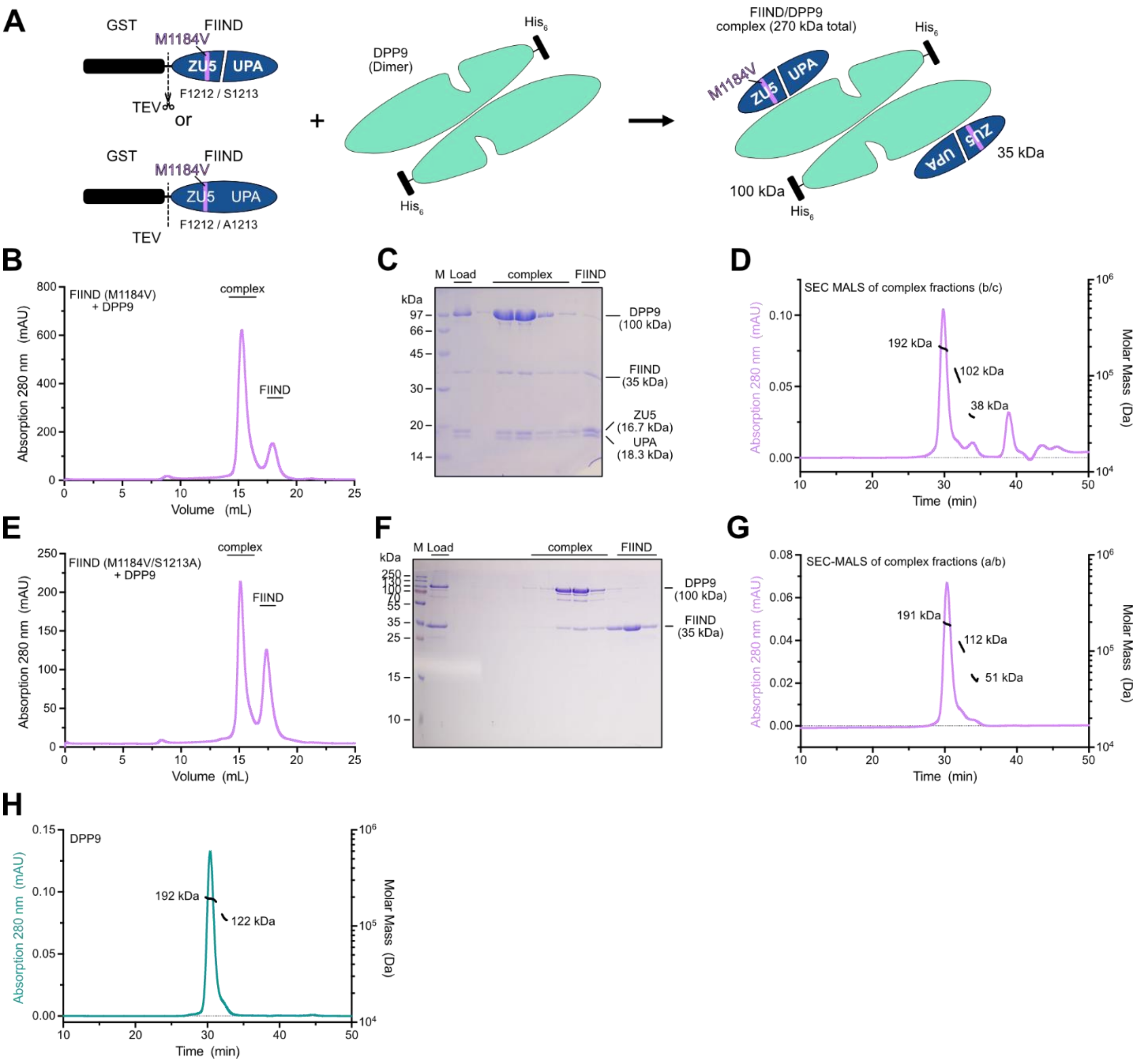

Figure S4. NLRP1 FIIND co-expression with DPP9.

A, Schematic of NLRP1-FIIND and DPP9 constructs used in co-expression experiments. *B*, Elution profile of NLRP1-FIIND (M1184V) co-expressed with DPP9 run on a Superose 6 size exclusion column. *C*, SDS-PAGE of the elution fractions from *B*. *D*, SEC-MALS analysis of co-purified NLRP1-FIIND(M1184V) and DPP9. The apparent molecular weight determined by light scattering for each peak is indicated in kDa. *E,* Elution profile of NLRP1-FIIND (M1184V/S1213A) co-expressed with DPP9 run on a Superose 6 size exclusion column. *F*, SDS-PAGE analysis of the elution fractions shown in *E*. *G*, SEC-MALS analysis of Peak 1 from the elution profile in *E*. *H*, SEC-MALS analysis of DPP9 alone. The majority of the protein elutes as a dimer with an apparent molecular weight of 192 kDa.

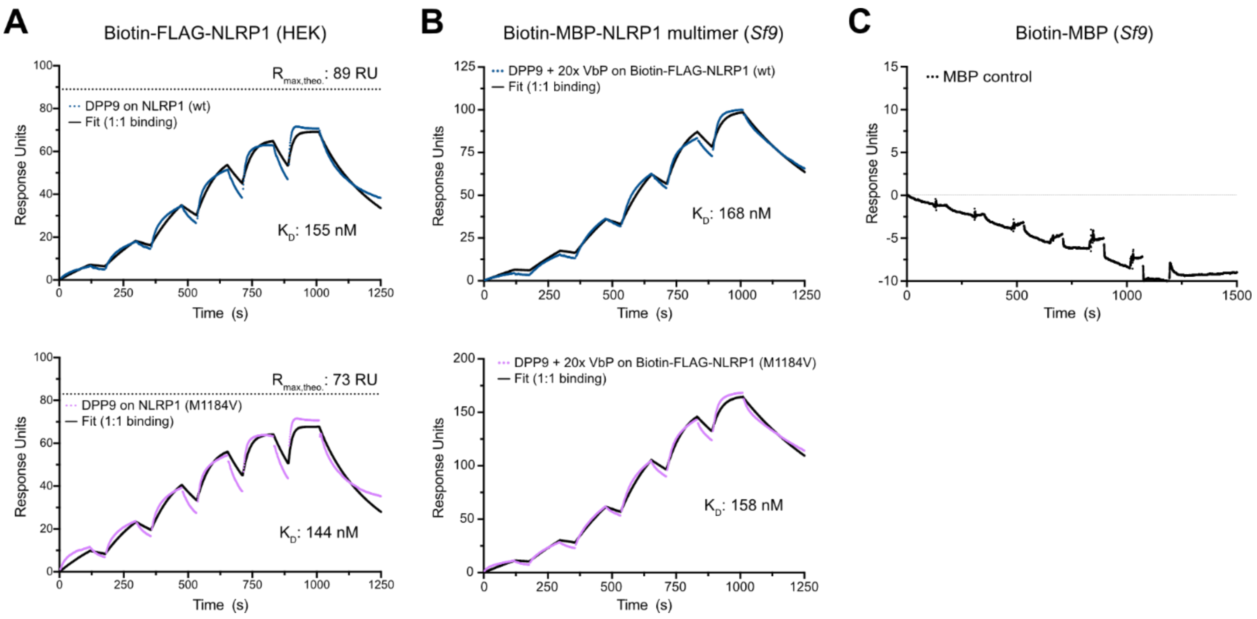

Figure S5. SPR control measurements of DPP9 binding to MBP-NLRP1 and MBP.
A, SPR analysis of DPP9 binding to immobilized full-length Biotin-FLAG-NLRP1 (wt or M1184V) purified from HEK293T cells. *B*-*C*, Single cycle kinetics SPR measurements of DPP9 on immobilized Biotin-MBP-NLRP1 (wt or M1184V) multimer or on Biotin-MBP (negative control). Kinetics on NLRP1 protein were recorded with the inhibitor Val-boroPro (VbP) present at a 20-fold excess in the DPP9 dilution series.
